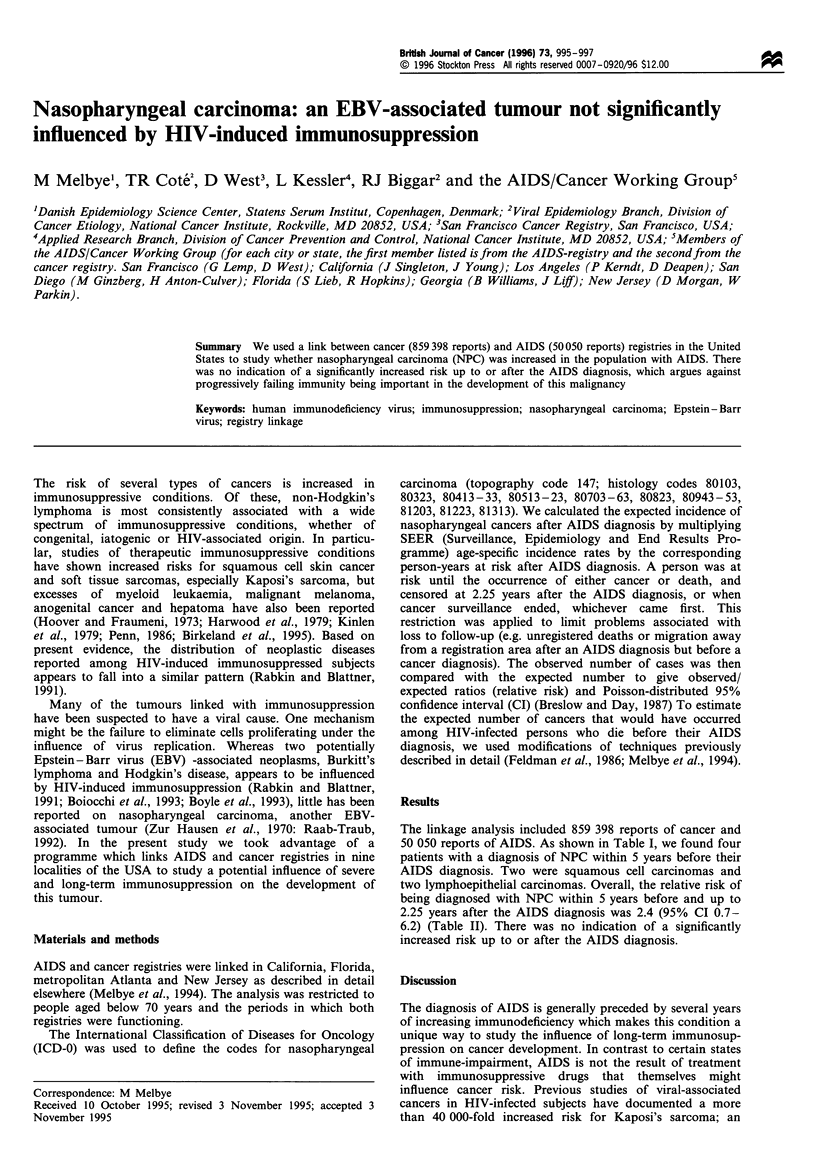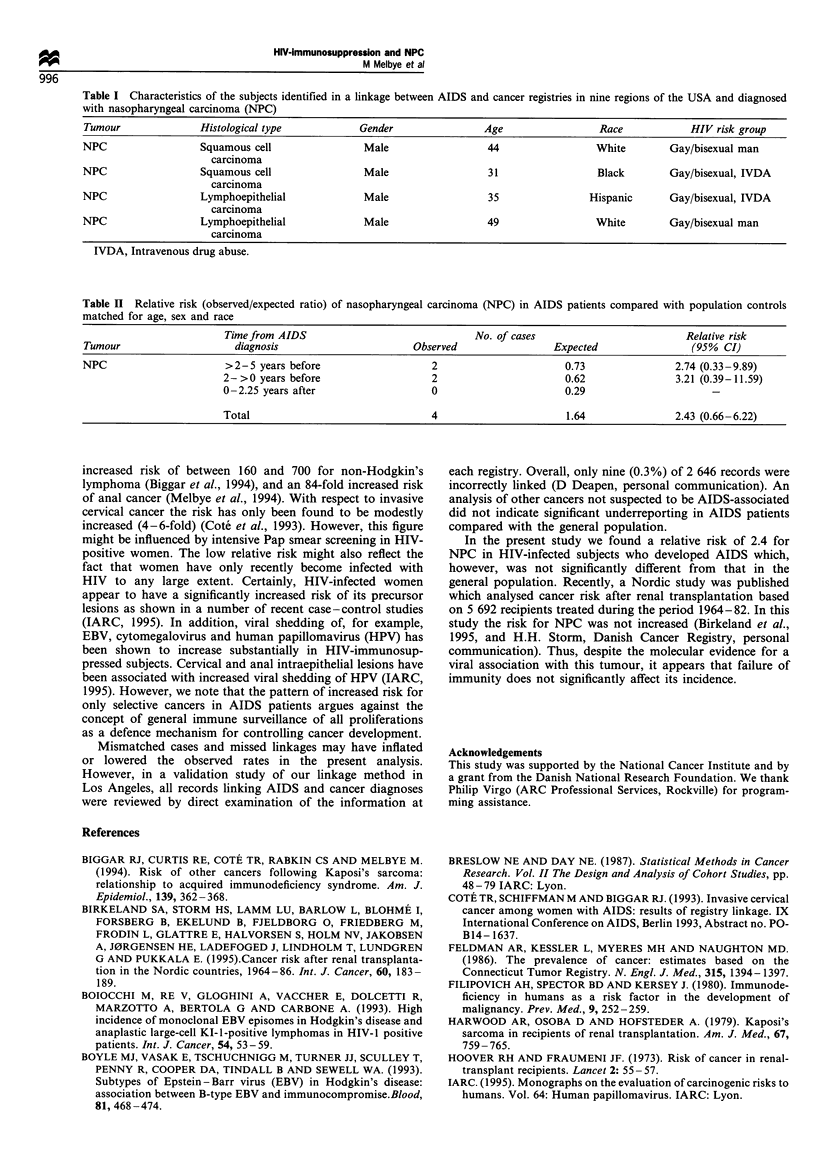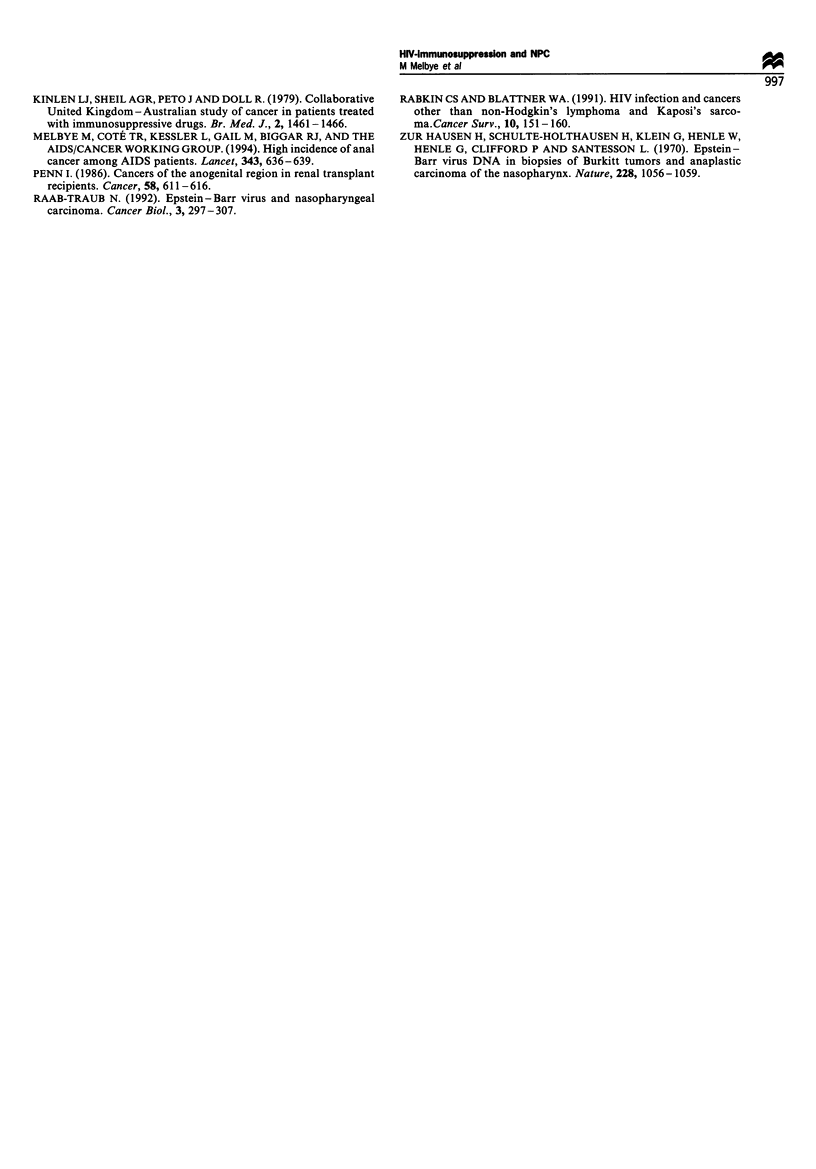# Nasopharyngeal carcinoma: an EBV-associated tumour not significantly influenced by HIV-induced immunosuppression. The AIDS/Cancer Working Group.

**DOI:** 10.1038/bjc.1996.194

**Published:** 1996-04

**Authors:** M. Melbye, T. R. Coté, D. West, L. Kessler, R. J. Biggar

**Affiliations:** Danish Epidemiology Science Center, Statens Serum Institut, Copenhagen, Denmark.

## Abstract

We used a link between cancer (859,398 reports) and AIDS (50,050 reports) registries in the United States to study whether nasopharyngeal carcinoma (NPC) was increased in the population with AIDS. There was no indication of a significantly increased risk up to or after the AIDS diagnosis, which argues against progressively failing immunity being important in the development of this malignancy.


British Journal of Cancer (1996) 73, 995-997
© 1996 Stockton Press All rights reserved 0007-0920/96 $12.00

### Nasopharyngeal carcinoma: an EBV-associated tumour not significantly influenced by HIV-induced immunosuppression

M Melbye[1], TR Coté[2], D West[3], L Kessler[4], RJ Biggar[2] and the AIDS/Cancer Working Group[5]

[1]*Danish Epidemiology Science Center, Statens Serum Institut, Copenhagen, Denmark;* [2]*Viral Epidemiology Branch, Division of Cancer Etiology, National Cancer Institute, Rockville, MD 20852, USA;* [3]*San Francisco Cancer Registry, San Francisco, USA;* [4]*Applied Research Branch, Division of Cancer Prevention and Control, National Cancer Institute, MD 20852, USA;* [5]*Members of the AIDS/Cancer Working Group (for each city or state, the first member listed is from the AIDS-registry and the second from the cancer registry. San Francisco (G Lemp, D West); California (J Singleton, J Young); Los Angeles (P Kerndt, D Deapen); San Diego (M Ginzberg, H Anton-Culver); Florida (S Lieb, R Hopkins); Georgia (B Williams, J Liff); New Jersey (D Morgan, W Parkin).*

**Summary** We used a link between cancer (859 398 reports) and AIDS (50 050 reports) registries in the United States to study whether nasopharyngeal carcinoma (NPC) was increased in the population with AIDS. There was no indication of a significantly increased risk up to or after the AIDS diagnosis, which argues against progressively failing immunity being important in the development of this malignancy

**Keywords:** human immunodeficiency virus; immunosuppression; nasopharyngeal carcinoma; Epstein-Barr virus; registry linkage

The risk of several types of cancers is increased in immunosuppressive conditions. Of these, non-Hodgkin's lymphoma is most consistently associated with a wide spectrum of immunosuppressive conditions, whether of congenital, iatogenic or HIV-associated origin. In particular, studies of therapeutic immunosuppressive conditions have shown increased risks for squamous cell skin cancer and soft tissue sarcomas, especially Kaposi's sarcoma, but excesses of myeloid leukaemia, malignant melanoma, anogenital cancer and hepatoma have also been reported (Hoover and Fraumeni, 1973; Harwood *et al.*, 1979; Kinlen *et al.*, 1979; Penn, 1986; Birkeland *et al.*, 1995). Based on present evidence, the distribution of neoplastic diseases reported among HIV-induced immunosuppressed subjects appears to fall into a similar pattern (Rabkin and Blattner, 1991).

Many of the tumours linked with immunosuppression have been suspected to have a viral cause. One mechanism might be the failure to eliminate cells proliferating under the influence of virus replication. Whereas two potentially Epstein-Barr virus (EBV) -associated neoplasms, Burkitt's lymphoma and Hodgkin's disease, appears to be influenced by HIV-induced immunosuppression (Rabkin and Blattner, 1991; Boiocchi *et al.*, 1993; Boyle *et al.*, 1993), little has been reported on nasopharyngeal carcinoma, another EBV-associated tumour (Zur Hausen *et al.*, 1970: Raab-Traub, 1992). In the present study we took advantage of a programme which links AIDS and cancer registries in nine localities of the USA to study a potential influence of severe and long-term immunosuppression on the development of this tumour.

### Materials and methods

AIDS and cancer registries were linked in California, Florida, metropolitan Atlanta and New Jersey as described in detail elsewhere (Melbye *et al.*, 1994). The analysis was restricted to people aged below 70 years and the periods in which both registries were functioning.

The International Classification of Diseases for Oncology (ICD-0) was used to define the codes for nasopharyngeal carcinoma (topography code 147; histology codes 80103, 80323, 80413-33, 80513-23, 80703-63, 80823, 80943-53, 81203, 81223, 81313). We calculated the expected incidence of nasopharyngeal cancers after AIDS diagnosis by multiplying SEER (Surveillance, Epidemiology and End Results Programme) age-specific incidence rates by the corresponding person-years at risk after AIDS diagnosis. A person was at risk until the occurrence of either cancer or death, and censored at 2.25 years after the AIDS diagnosis, or when cancer surveillance ended, whichever came first. This restriction was applied to limit problems associated with loss to follow-up (e.g. unregistered deaths or migration away from a registration area after an AIDS diagnosis but before a cancer diagnosis). The observed number of cases was then compared with the expected number to give observed/ expected ratios (relative risk) and Poisson-distributed 95% confidence interval (CI) (Breslow and Day, 1987) To estimate the expected number of cancers that would have occurred among HIV-infected persons who die before their AIDS diagnosis, we used modifications of techniques previously described in detail (Feldman *et al.*, 1986; Melbye *et al.*, 1994).

### Results

The linkage analysis included 859 398 reports of cancer and 50 050 reports of AIDS. As shown in Table I, we found four patients with a diagnosis of NPC within 5 years before their AIDS diagnosis. Two were squamous cell carcinomas and two lymphoepithelial carcinomas. Overall, the relative risk of being diagnosed with NPC within 5 years before and up to 2.25 years after the AIDS diagnosis was 2.4 (95% CI 0.7-6.2) (Table II). There was no indication of a significantly increased risk up to or after the AIDS diagnosis.

### Discussion

The diagnosis of AIDS is generally preceded by several years of increasing immunodeficiency which makes this condition a unique way to study the influence of long-term immunosuppression on cancer development. In contrast to certain states of immune-impairment, AIDS is not the result of treatment with immunosuppressive drugs that themselves might influence cancer risk. Previous studies of viral-associated cancers in HIV-infected subjects have documented a more than 40 000-fold increased risk for Kaposi's sarcoma; an

Correspondence: M Melbye
Received 10 October 1995; revised 3 November 1995; accepted 3 November 1995

Table I Characteristics of the subjects identified in a linkage between AIDS and cancer registries in nine regions of the USA and diagnosed with nasopharyngeal carcinoma (NPC)

| *Tumour* | *Histological type* | *Gender* | *Age* | *Race* | *HIV risk group* |
|---|---|---|---|---|---|
| NPC | Squamous cell carcinoma | Male | 44 | White | Gay/bisexual man |
| NPC | Squamous cell carcinoma | Male | 31 | Black | Gay/bisexual, IVDA |
| NPC | Lymphoepithelial carcinoma | Male | 35 | Hispanic | Gay/bisexual, IVDA |
| NPC | Lymphoepithelial carcinoma | Male | 49 | White | Gay/bisexual man |

IVDA, Intravenous drug abuse.

Table II Relative risk (observed/expected ratio) of nasopharyngeal carcinoma (NPC) in AIDS patients compared with population controls matched for age, sex and race

| *Tumour* | *Time from AIDS diagnosis* | *No. of cases* *Observed* | *Expected* | *Relative risk (95% CI)* |
|---|---|---|---|---|
| NPC | >2–5 years before | 2 | 0.73 | 2.74 (0.33–9.89) |
| | 2–>0 years before | 2 | 0.62 | 3.21 (0.39–11.59) |
| | 0–2.25 years after | 0 | 0.29 | – |
| | Total | 4 | 1.64 | 2.43 (0.66–6.22) |

increased risk of between 160 and 700 for non-Hodgkin's lymphoma (Biggar *et al.*, 1994), and an 84-fold increased risk of anal cancer (Melbye *et al.*, 1994). With respect to invasive cervical cancer the risk has only been found to be modestly increased (4–6-fold) (Coté *et al.*, 1993). However, this figure might be influenced by intensive Pap smear screening in HIV-positive women. The low relative risk might also reflect the fact that women have only recently become infected with HIV to any large extent. Certainly, HIV-infected women appear to have a significantly increased risk of its precursor lesions as shown in a number of recent case–control studies (IARC, 1995). In addition, viral shedding of, for example, EBV, cytomegalovirus and human papillomavirus (HPV) has been shown to increase substantially in HIV-immunosuppressed subjects. Cervical and anal intraepithelial lesions have been associated with increased viral shedding of HPV (IARC, 1995). However, we note that the pattern of increased risk for only selective cancers in AIDS patients argues against the concept of general immune surveillance of all proliferations as a defence mechanism for controlling cancer development.

Mismatched cases and missed linkages may have inflated or lowered the observed rates in the present analysis. However, in a validation study of our linkage method in Los Angeles, all records linking AIDS and cancer diagnoses were reviewed by direct examination of the information at each registry. Overall, only nine (0.3%) of 2 646 records were incorrectly linked (D Deapen, personal communication). An analysis of other cancers not suspected to be AIDS-associated did not indicate significant underreporting in AIDS patients compared with the general population.

In the present study we found a relative risk of 2.4 for NPC in HIV-infected subjects who developed AIDS which, however, was not significantly different from that in the general population. Recently, a Nordic study was published which analysed cancer risk after renal transplantation based on 5 692 recipients treated during the period 1964–82. In this study the risk for NPC was not increased (Birkeland *et al.*, 1995, and H.H. Storm, Danish Cancer Registry, personal communication). Thus, despite the molecular evidence for a viral association with this tumour, it appears that failure of immunity does not significantly affect its incidence.

**Acknowledgements**
This study was supported by the National Cancer Institute and by a grant from the Danish National Research Foundation. We thank Philip Virgo (ARC Professional Services, Rockville) for programming assistance.

### References

BIGGAR RJ, CURTIS RE, COTÉ TR, RABKIN CS AND MELBYE M. (1994). Risk of other cancers following Kaposi's sarcoma: relationship to acquired immunodeficiency syndrome. *Am. J. Epidemiol.*, **139**, 362–368.

BIRKELAND SA, STORM HS, LAMM LU, BARLOW L, BLOHMÉ I, FORSBERG B, EKELUND B, FJELDBORG O, FRIEDBERG M, FRODIN L, GLATTRE E, HALVORSEN S, HOLM NV, JAKOBSEN A, JØRGENSEN HE, LADEFOGED J, LINDHOLM T, LUNDGREN G AND PUKKALA E. (1995).Cancer risk after renal transplantation in the Nordic countries, 1964–86. *Int. J. Cancer*, **60**, 183–189.

BOIOCCHI M, RE V, GLOGHINI A, VACCHER E, DOLCETTI R, MARZOTTO A, BERTOLA G AND CARBONE A. (1993). High incidence of monoclonal EBV episomes in Hodgkin's disease and anaplastic large-cell KI-1-positive lymphomas in HIV-1 positive patients. *Int. J. Cancer*, **54**, 53–59.

BOYLE MJ, VASAK E, TSCHUCHNIGG M, TURNER JJ, SCULLEY T, PENNY R, COOPER DA, TINDALL B AND SEWELL WA. (1993). Subtypes of Epstein–Barr virus (EBV) in Hodgkin's disease: association between B-type EBV and immunocompromise.*Blood*, **81**, 468–474.

BRESLOW NE AND DAY NE. (1987). *Statistical Methods in Cancer Research. Vol. II The Design and Analysis of Cohort Studies*, pp. 48–79 IARC: Lyon.

COTÉ TR, SCHIFFMAN M AND BIGGAR RJ. (1993). Invasive cervical cancer among women with AIDS: results of registry linkage. IX International Conference on AIDS, Berlin 1993, Abstract no. PO-B14–1637.

FELDMAN AR, KESSLER L, MYERES MH AND NAUGHTON MD. (1986). The prevalence of cancer: estimates based on the Connecticut Tumor Registry. *N. Engl. J. Med.*, **315**, 1394–1397.

FILIPOVICH AH, SPECTOR BD AND KERSEY J. (1980). Immunodeficiency in humans as a risk factor in the development of malignancy. *Prev. Med.*, **9**, 252–259.

HARWOOD AR, OSOBA D AND HOFSTEDER A. (1979). Kaposi's sarcoma in recipients of renal transplantation. *Am. J. Med.*, **67**, 759–765.

HOOVER RH AND FRAUMENI JF. (1973). Risk of cancer in renal-transplant recipients. *Lancet* **2**: 55–57.

IARC. (1995). Monographs on the evaluation of carcinogenic risks to humans. Vol. 64: Human papillomavirus. IARC: Lyon.

KINLEN LJ, SHEIL AGR, PETO J AND DOLL R. (1979). Collaborative United Kingdom–Australian study of cancer in patients treated with immunosuppressive drugs. *Br. Med. J.*, **2**, 1461–1466.

MELBYE M, COTÉ TR, KESSLER L, GAIL M, BIGGAR RJ, AND THE AIDS/CANCER WORKING GROUP. (1994). High incidence of anal cancer among AIDS patients. *Lancet*, **343**, 636–639.

PENN I. (1986). Cancers of the anogenital region in renal transplant recipients. *Cancer*, **58**, 611–616.

RAAB-TRAUB N. (1992). Epstein–Barr virus and nasopharyngeal carcinoma. *Cancer Biol.*, **3**, 297–307.

RABKIN CS AND BLATTNER WA. (1991). HIV infection and cancers other than non-Hodgkin's lymphoma and Kaposi's sarcoma.*Cancer Surv.*, **10**, 151–160.

ZUR HAUSEN H, SCHULTE-HOLTHAUSEN H, KLEIN G, HENLE W, HENLE G, CLIFFORD P AND SANTESSON L. (1970). Epstein–Barr virus DNA in biopsies of Burkitt tumors and anaplastic carcinoma of the nasopharynx. *Nature*, **228**, 1056–1059.